# Deep brain stimulation for essential tremor versus essential tremor plus: should we target the same spot in the thalamus?

**DOI:** 10.3389/fnhum.2023.1271046

**Published:** 2023-10-31

**Authors:** Cherry H. Yu, Daniel H. Lench, Christine Cooper, Nathan C. Rowland, Istvan Takacs, Gonzalo Revuelta

**Affiliations:** ^1^Department of Neurology, Medical University of South Carolina, Charleston, SC, United States; ^2^Ralph H. Johnson VA Medical Center, Charleston, SC, United States; ^3^Department of Neurosurgery, Medical University of South Carolina, Charleston, SC, United States

**Keywords:** essential tremor, essential tremor plus, deep brain stimulation, DBS target, VIM

## Abstract

**Background:**

Although ET is a phenomenologically heterogeneous condition, thalamic DBS appears to be equally effective across subtypes. We hypothesized stimulation sites optimized for individuals with essential tremor (ET) would differ from individuals with essential tremor plus syndrome (ET-plus). We examined group differences in optimal stimulation sites within the ventral thalamus and their overlap of with relevant white matter tracts. By capturing these differences, we sought to determine whether ET subtypes are associated with anatomically distinct neural pathways.

**Methods:**

A retrospective chart review was conducted on ET patients undergoing VIM DBS at MUSC between 01/2012 and 02/2022. Clinical, demographic, neuroimaging, and DBS stimulation parameter data were collected. Clinical characteristics and pre-DBS videos were reviewed to classify ET and ET-plus cohorts. Patients in ET-plus cohorts were further divided into ET with dystonia, ET with ataxia, and ET with others. DBS leads were reconstructed using Lead-DBS^1^ and the volume of tissue activated (VTA) overlap was performed using normative connectomes. Tremor improvement was measured by reduction in a subscore of tremor rating scale (TRS) post-DBS lateralized to the more affected limb.

**Results:**

Sixty-eight ET patients were enrolled after initial screening, of these 10 ET and 24 ET-plus patients were included in the final analyses. ET group had an earlier age at onset (*p* = 0.185) and underwent surgery at a younger age (*p* = 0.096). Both groups achieved effective tremor control. No significant differences were found in lead placement or VTA overlap within ventral thalamus. The VTA center of gravity (COG) in the ET-plus cohort was located dorsal to that of the ET cohort. No significant differences were found in VTA overlap with the dentato-rubral-thalamic (DRTT) tracts or the ansa lenticularis. Dystonia was more prevalent than ataxia in the ET-plus subgroups (*n* = 18 and *n* = 5, respectively). ET-plus with dystonia subgroup had a more medial COG compared to ET-plus with ataxia.

**Conclusion:**

VIM DBS therapy is efficacious in patients with ET and ET-plus. There were no significant differences in optimal stimulation site or VTA overlap with white-matter tracts between ET, ET-plus and ET-plus subgroups.

## Introduction

Essential tremor (ET) is one of the most common movement disorders, affecting up to 1% of the world population, and its prevalence increases up to 4–5% in the elderly population (Louis and Ferreira, 2010; Clark and Louis, 2018; Shanker, 2019). Symptoms of ET often emerge in a bimodal distribution across age groups, affecting people in their second to third decades or after the fifth or sixth decades of life (Louis and Ferreira, 2010; Clark and Louis, 2018; Haubenberger and Hallett, 2018). ET is characterized by upper limb action and/or postural tremor and can affect areas such as the head, voice, and lower limbs. ET is typically inherited in an autosomal dominant fashion with reduced penetrance (Bhatia et al., 2018; Clark and Louis, 2018; Haubenberger and Hallett, 2018). The pathophysiology of ET remains incompletely understood but there is increasing neuroimaging and postmortem evidence of cerebellar pathology (Kuo et al., 2011; Bhalsing et al., 2013; Gionco et al., 2021; Holtbernd and Shah, 2021; Pan and Kuo, 2022). Specifically, the cerebello-thalamo-cortical loop plays a major role in ET tremorgenesis (Lenka et al., 2017; Nicoletti et al., 2020).

In 2018, the Tremor Task Force from the Movement Disorders Society (MDS) revised the consensus statement for tremor classification and introduced the diagnosis of Essential tremor plus (ET-plus). ET-plus is defined as “tremor with characteristics of ET and additional neurologic signs of uncertain significance such as impaired tandem gait, questionable dystonic posturing, memory impairment, or other mild neurologic signs of unknown significance that do not suffice to make an additional syndrome classification or diagnosis” (Bhatia et al., 2018). The updated criteria emphasized the heterogeneity of ET syndrome, and the designation of ET-plus aimed to create a more refined patient selection in clinical research.

Propranolol and primidone are the most common first line treatments and may achieve up to 70% tremor reduction when used in combination (Koller and Royse, 1986; Ferreira et al., 2019). However, pharmacological treatment of ET and ET-plus is often inadequate and limited by undesirable side effects. Prior literature reported up to 50% of patients discontinuing medical therapy due to intolerance (Louis et al., 2010). Deep brain stimulation (DBS) has been the mainstay treatment for medically refractory tremor since its initial approval in 1997 (Iorio-Morin et al., 2020; Wong et al., 2020). Stimulation to ventralis intermedius nucleus (VIM) of the thalamus has demonstrated excellent efficacy in the treatment of medically refractory tremor, achieving up to 66–80% tremor reduction with sustained long term efficacy (Zhang et al., 2010; Dallapiazza et al., 2019; Paschen et al., 2019). Several targets have been explored for optimal tremor reduction including the posterior subthalamic area (PSA) and zona inserta (ZI) (Wong et al., 2020; Chandra et al., 2022). VIM remains the most common target as it provides better long-term efficacy, and stimulation to deeper targets may result in higher rates of stimulation induced ataxia and dysarthria due to involvement of the cerebellothalmic tracts (Iorio-Morin et al., 2020; Wong et al., 2020; Kremer et al., 2021; Chandra et al., 2022). Stimulation to the dentato-rubral-thalamic tracts (DRTT) with projected connections to the primary motor and supplementary motor cortices have been implicated to produce the most efficacy in tremor reduction (Iorio-Morin et al., 2020; Wong et al., 2020; Middlebrooks et al., 2021; Chandra et al., 2022). The DRTT connects the cerebellum to the thalamus with receiving fibers primarily in the VIM, and consists of both decussating (DRTT) and non-decussating (nDRTT) fibers (Gallay et al., 2008). Adjacent stimulation to the pallidothalamic tracts (ansa lenticularis and fasciculus lenticularis), which originate from the globus pallidus interna (GPi) with implications in the treatment of dystonia, has also demonstrated tremor efficacy (Gallay et al., 2008; Iorio-Morin et al., 2020; Horisawa et al., 2021).

Two recent studies have demonstrated similar efficacy in treatment of ET and ET-plus with VIM DBS (Steffen et al., 2020; Gilmour et al., 2021), but whether ET and ET-plus represent distinct clinical entities with varying underlying pathophysiology remains unexplored. We hypothesized that the effective stimulation site within the ventral thalamus may differ between ET and ET-plus subtypes. We aimed to focus specifically on two phenomenologically distinct ET-plus subtypes: ET-plus with dystonia and ET-plus with ataxia. We also hypothesized that the volume of tissue activated (VTA) overlaps with adjacent white matter tracts of ET subtypes would also differ. The pathophysiology of dystonia is hypothesized to involve both the cerebello-thalamo-cortical and the basal ganglia-thalamo-cortical networks. A recent study by Tsuboi et al. demonstrated slightly different functional and structural connectivity between dystonic and essential tremor (Middlebrooks et al., 2021). Animal and small cerebellar DBS studies have demonstrated aberrant hyperexcitability of the deep cerebellar nuclei as the potential culprit for symptoms of ataxia and kinetic tremor (Tai and Tseng, 2022). By capturing differences in tract engagement of VTA overlap to the optimal stimulation site in ET subtypes, we sought to determine whether these subtypes are pathophysiologically distinct from ET.

## Materials and methods

We performed a retrospective chart and video review of all patients who underwent VIM DBS for ET and ET-plus at the Medical University of South Carolina between 01/2012 and 02/2022. ET was defined based on the tremor classification as “isolated tremor syndrome of bilateral upper limb action tremor, at least 3 years duration, with or without tremor in other locations (e.g., head, voice or lower limbs), and the absence of other neurological signs, such as dystonia, ataxia or parkinsonism” (Bhatia et al., 2018). ET-plus was defined as “tremor with the characteristics of ET and additional neurological signs of uncertain significance such as impaired tandem gait, questionable dystonic posturing, memory impairment, or other mild neurological signs of unknown significance that do not suffice to make an additional syndrome classification or diagnosis” (Bhatia et al., 2018). Patients in the ET-plus cohort were further divided into ET-plus with dystonia, ET-plus with ataxia, and ET-plus with other based on characteristics observed in pre-operative videos. Inclusion criteria were: (i) clinical diagnosis of ET or ET-plus at the time of DBS implantation; (ii) DBS insertion in the VIM nucleus of the thalamus; (iii) pre-operative video available for review, (iv) preoperative brain MRI and postoperative CT data available. Exclusion criteria included were: (i) missing stimulation parameters; (ii) lack of efficacy data; (iii) suboptimal quality to preoperative MRI or postoperative CT images; (iv) significant surgical complications resulting in the removal of device or permanent neurologic deficits; and (v) concomitant comorbidities that would potentially confound the delineation of diagnosis or outcome measure (i.e., functional neurological disorder, history of CNS infection or traumatic brain injury).

Baseline clinical characteristics collected included: gender, race, ethnicity, handedness, age at onset of ET or ET-plus, more effected limb, age at surgery, family history of tremor, tremor characteristics (body distribution, activation conditions, symmetry), previous treatments, significant comorbidities, additional neurological signs of uncertain significance (e.g., dystonia, rigidity, bradykinesia, myoclonus, mild cognitive impairment identified during presurgical neuropsychologic evaluation, and/or impaired tandem gait).

Tremor characteristics to appropriately classify patients as having ET or ET-plus were extracted independently from pre-operative videos by one movement disorders specialist (CHY), with supplementation from paper charts for aspects of the examination not filmed (e.g., mild cognitive impairment, subtle abnormal posturing and/or irregular head tremor, impaired tandem gait, and/or subtle limb ataxia).

Tremor severity was evaluated using the Fahn-Tolosa-Marin Tremor Rating Scale (TRS) motor scores (items 1–14) where higher scores indicated worse tremor. The primary outcome measure was a reduction in the pre- to post-operative tremor subscores, lateralized to the more affected limb before DBS implantation (Items 1–5, 7–8 for the right hand or items 1–4, 6–7, and 9 for the left hand).

### Standard perioperative procedures

Prior to implantation, DBS candidacy was determined by a multidisciplinary team including movement disorders neurologists, neurosurgeons, neuropsychologists and speech and language pathologists. Preoperatively, a standard stereotactic targeting MRI was performed on a 3 T Siemens scanner (Siemens Healthineers AG), which included a high-resolution T1-weighted magnetization-prepared rapid gradient-echo (MP-RAGE) sequence optimized for differentiation of gray and white matter. The VIM was targeted using standard AC-PC coordinates (x = 11.5 lateral + half of the width of the third ventricle, y = anterior to posterior commissure (PC) by 20% of anterior commissure (AC) to posterior commissure line length, and z = along AC-PC line all measured in millimeters). DBS leads (Medtronic 3,389, Abbott 6,172, or Boston Scientific Vercise) were implanted under local anesthesia with additional guidance obtained with intraoperative microelectrode recordings and macrostimulation testing. We aimed to place the distal contact at the ventral border of the VIM. The latest available pulse generators were implanted. Three to four weeks after implantation, patients underwent postoperative CT scans and a monopolar review to evaluate the initial tremor-suppressing effects and adverse effects of each contact. A postoperative CT was obtained on Siemens helical CT scanner (Siemens Healthineers AG) with an in-plane resolution of 0.5 × 0.5 mm and slice thickness of 1 mm. Following monopolar review, patients had regular follow up visits to optimize their DBS settings. Clinical follow up period was defined by months after initial programming session with monopolar review.

### DBS electrode localization

Lead localization was performed using Lead-DBS software (V2.6). Raw pre-operative MRI scans and post-operative CT scans were converted to NIFTI file formats using dcm2niix (Li et al., 2016). Post-operative CT scans were then co-registered to either a T1 or T2 pre-operative scan (depending on acquisition quality and availability) using a two-stage linear registration as implemented in Advanced Normalization Tools (Avants et al., 2008).^2^ Other pre-operative MRI scans (e.g., PD, FGAITR, FLAIR) were co-registered with the T1 or T2 scan using SPM12.^3^ Normalization of pre-operative and post-operative images to the MNI_ICBM_2009b_NLIN_ASYM template space was performed using the FNIRT approach as implemented in the FMRIB Software Library.^4^ Image co-registration and normalization quality was manually reviewed. In the case of poorly co-registered images, volume registrations were redone using an alternative approach to maximize registration quality. DBS electrode localizations were corrected for brainshift in postoperative acquisitions by applying a refined affine transform calculated between pre- and postoperative acquisitions that were restricted to a subcortical area of interest as implemented in the brainshift-correction module of Lead-DBS software (see text footnote 1). DBS-Electrodes were manually localized based on post-operative acquisitions using a tool specifically designed for this task. 3D visualization of data was performed using the DISTAL atlas. Volume of tissue activated (VTA) were created by using stable, clinically programmed stimulation parameters determined during outpatient programming visits. VTAs were estimated in patient space using the SimBio/FieldTrip model as implemented in Lead-DBS and normalized into the MNI template space for further analyses (Horn et al., 2017). VTAs which were estimated in the right hemisphere were binarized and flipped to the left hemisphere to create VTA overlap maps in MNI space. Overlap maps were calculated by taking the sum of the binarized VTAs. Each lead was treated as an independent data point, with a total of 12 leads and VTAs reconstructed in the ET group and a total of 31 leads and VTAs reconstructed in the ET-plus group.

### VTA center of gravity and fiber pathway analysis

#### Center of gravity analysis

To summarize differences in the anatomical location of VTA’s within the ET and ET-plus groups, the center of gravity (COG) coordinates of the VTAs were calculated using FSL. The COG coordinate is the average location of VTA coordinates, weighted by the number of participants with VTAs at a given coordinate.

#### Fiber pathway analysis

To determine group differences in fiber activation between groups the overlap between VTAs and normative fiber pathways were calculated for each patient. The dentato-rubral-thalamic tract (DRTT), non-decussating dentato-rubral-thalamic tract (nDRTT) and ansa lenticularis fiber pathways were defined using a previously published diffusion tractography provided as an atlas in Lead-DBS (Middlebrooks et al., 2020). Two-sample t-tests were used to compare the number of overlapping voxels in the ET vs. the ET-plus group and considered significant if *p* < 0.05.

### Statistical analysis of demographics and clinical data

Statistical analyses were performed using R (R version 4.2.2). Differences between groups at baseline in demographics and clinical data were assessed using one-way ANOVA. A *p*-value of less than 0.05 was considered significant.

## Results

Sixty-eight ET patients who underwent VIM DBS at MUSC between 1/2012 to 2/2022 were included after initial screening with appropriate pre-operative MRI and post-operative CT imaging data. Thirty-four of these patients were excluded. Of the 34 patients included, 10 patients (29%) were characterized as ET, and 24 patients (71%) were ET-plus. Of note, 21 patients (87.5%) were re-classified as from ET to ET-plus. About 40% (*n* = 4) of the ET cohort and 38% (*n* = 9) of the ET-plus cohort underwent unilateral VIM DBS. The baseline demographics and clinical data of ET and ET-plus cohorts are summarized in Table 1. Within the ET-plus cohort, 18 patients were subcategorized as ET-plus with dystonia (75%), 5 patients as ET-plus with ataxia (21%), and 1 patient characterized as ET with other (parkinsonism) (4%). The individual data are provided in Supplementary Table S1.

**Table 1:** Baseline characteristics and DBS outcome.

	ET	ET-Plus	*P*
N	10	24	
Age at symptom onset, years	34.20 ± 19.85	40.79 ± 18.99	0.185
Age at DBS surgery, years	68.1 ± 7.40	71.5 ± 5.76	0.096
Gender (%Female)	30%	46%	
Race (%White)	100%	83%	
Family history of tremor (%Yes)	70%	71%	
Average follow up length, months	46.90 ± 28.12	31.58 ± 20.77	**0.044**
Baseline TRS, total	53.3 ± 8.60	53.4 ± 17	0.496
TRS unilateral pre-DBS (Items 1–5, 7–8 for right hand or items 1–4, 7 and 9 for left hand)	9.00 ± 9.73	10.09 ± 4.63	0.450
TRS unilateral post-DBS (Items 1–5, 7–8 for right hand or items 1–4, 6–7 and 9 for left hand)	(*N* = 8) 0.375 ± 0.52	(*N* = 16) 2.50 ± 1.67	**0.001**
TRS follow up period, months	(*N* = 8) 11.89 ± 6.41	(*N* = 16) 11.81 ± 9.28	0.491
TRS reduction, average	(*N* = 8) 82%	(*N* = 16) 70%	0.154

Data represented as mean ± standard deviation. Statistical analysis was performed using independent *t*-test. *p* < 0.05 is considered significant and shown in bold.

ET patients had younger mean age at onset (age 34 for ET, and age 41 for ET-plus, *p* = 0.185) and underwent DBS surgery at a younger age (age 68 for ET and age 72 for ET-plus, *p* = 0.096). They had a significantly longer average length at follow up (47 months for ET and 32 months for ET-plus, *p* = 0.044). There was no significant difference between baseline tremor severity as measured by pre-DBS total TRS or unilateral TRS lateralized to the more affected limb. The ET cohort has a significantly lower post-DBS unilateral TRS lateralized to the more affected limb (*p* = 0.001). VIM DBS stimulation improved the contralateral TRS tremor sub scores for both ET and ET-plus cohorts [Table 1; average 82 and 70% for ET (*n* = 8) and ET-plus (*n* = 16) respectively, *p* = 0.154]. Pre- and post-DBS TRS scores were obtained approximately within 12 months for both ET and ET-plus cohorts. Additional characteristics including short term (1–2 years post-operatively) and final stimulation parameters are included in Supplementary Table S1.

### Volume of tissue activated analysis

**Figure 1:**
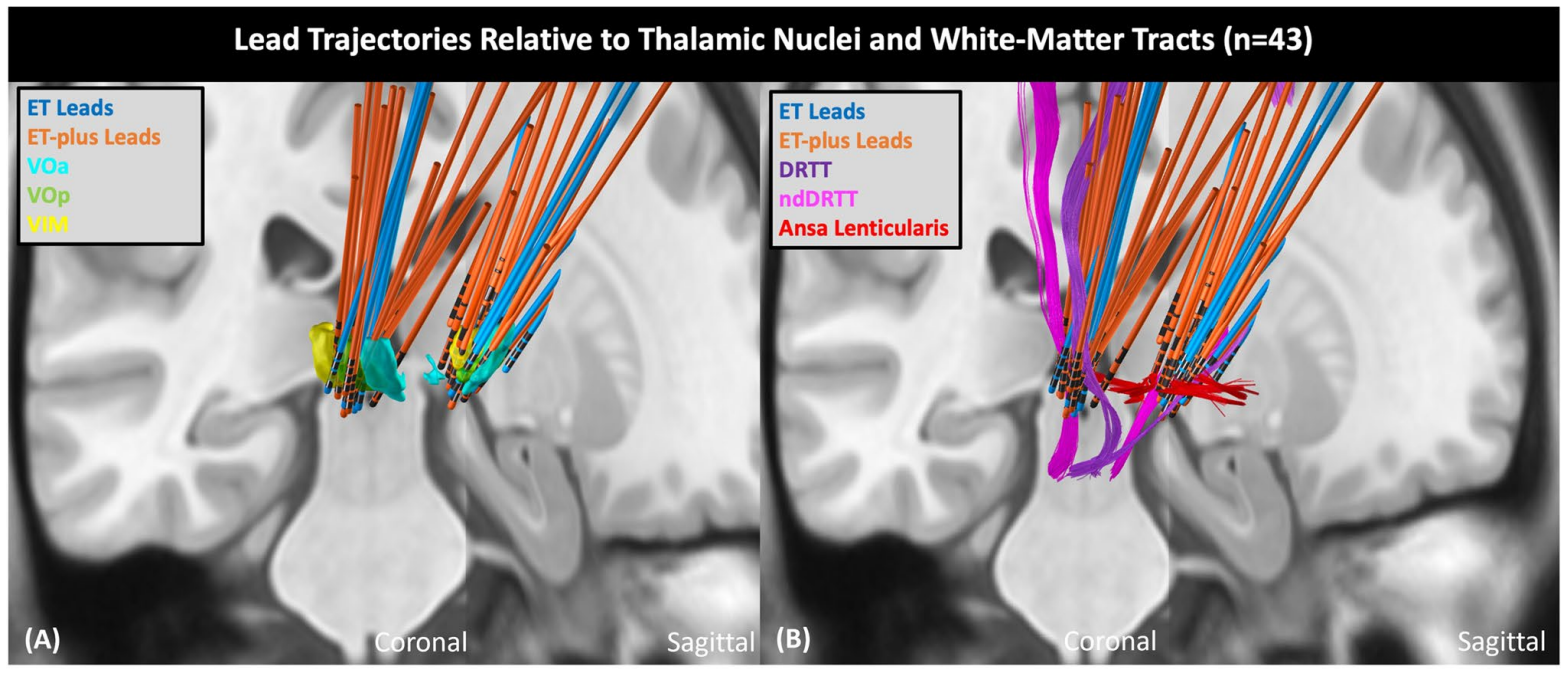
ET and ET-plus lead trajectories relative to thalamic nuclei and white matter tracts. **(A)** Shows DBS lead trajectories relative to thalamic nuclei in coronal (left) plane and sagittal (right) plane. **(B)** Shows DBS lead trajectories relative to adjacent white matter tracts. VOa, ventralis oralis anterior nucleus; VOp, ventralis oralis posterior nucleus; nDRTT, non-decussating dentato-rubral-thalamic tract.

**Figure 2:**
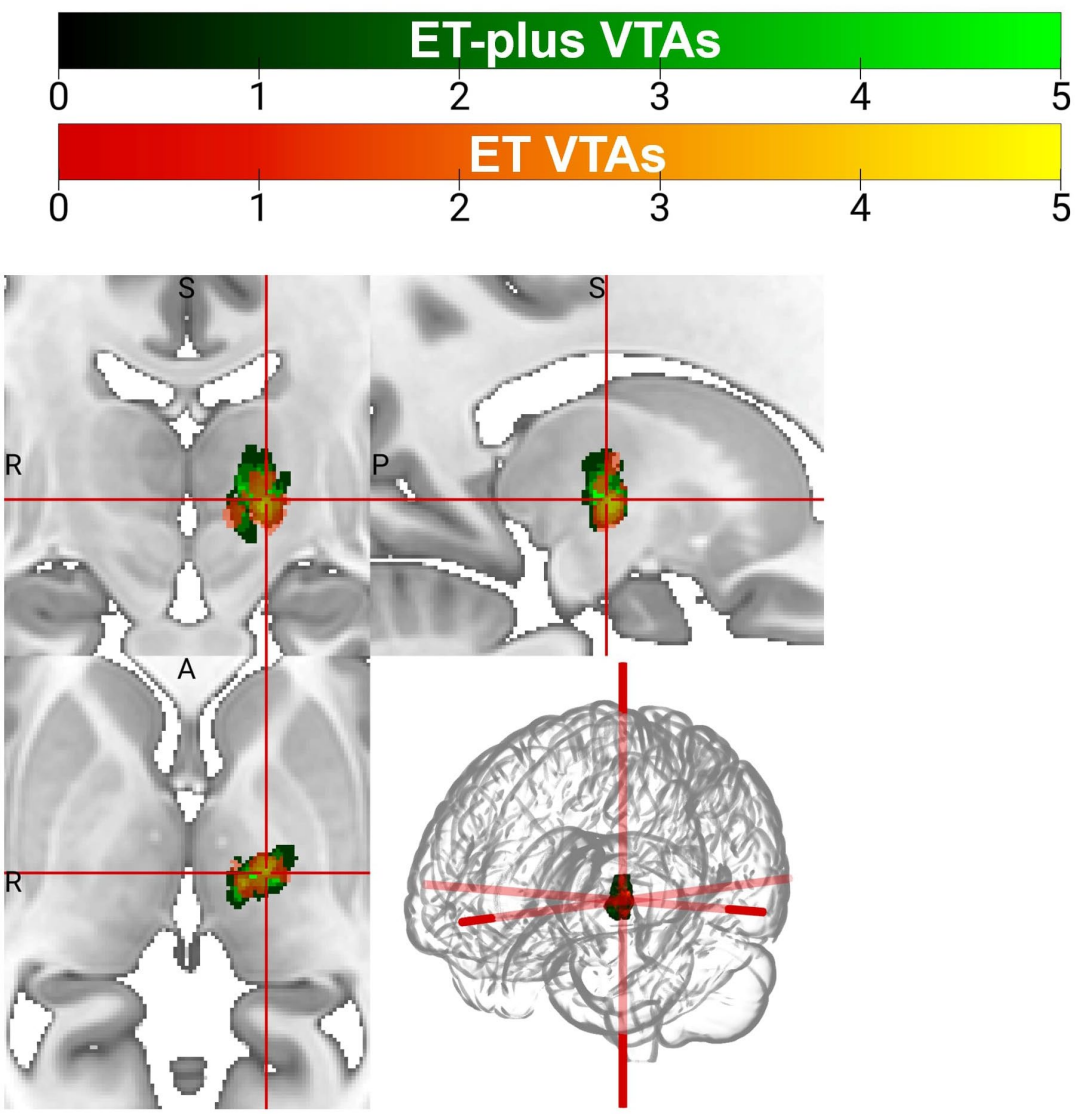
Volume of tissue activated (VTA) heatmap for patients with ET (orange) and ET-plus (green).

**Figure 3:**
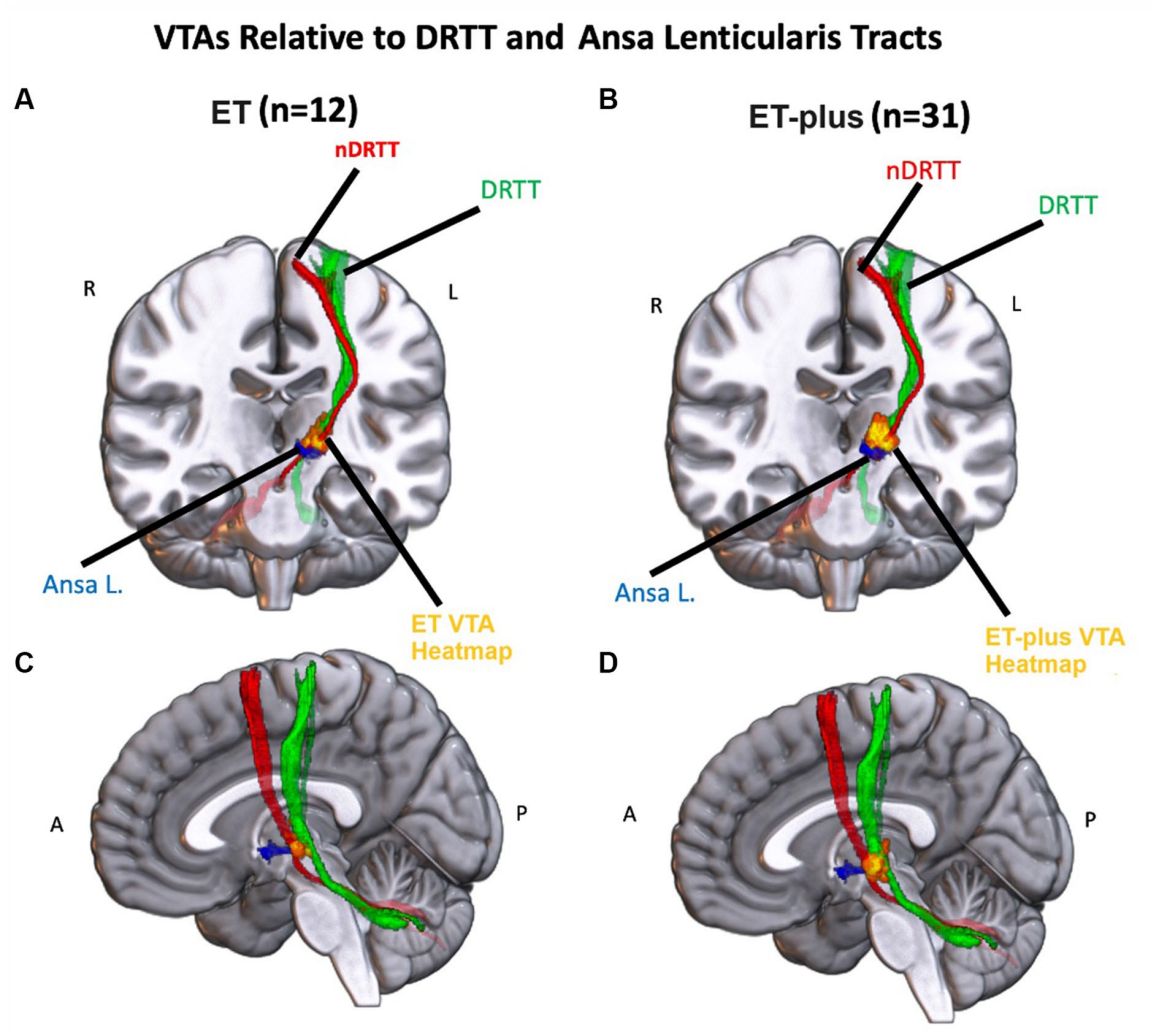
Left: Heatmap of the ET VTAs (red-yellow) relative to the nDRTT (red), the DRTT (green) and the Ansa Lenticularis (blue) in a coronal **(A)** and sagittal **(C)** view of an MNI-152 brain. Right: Heatmap of the ET+ VTAs (red-yellow) relative to the nDRTT (red), the DRTT (green) and the Ansa Lenticularis (blue) in a coronal **(B)** and sagittal **(D)** view of an MNI-152 brain.

**Figure 4:**
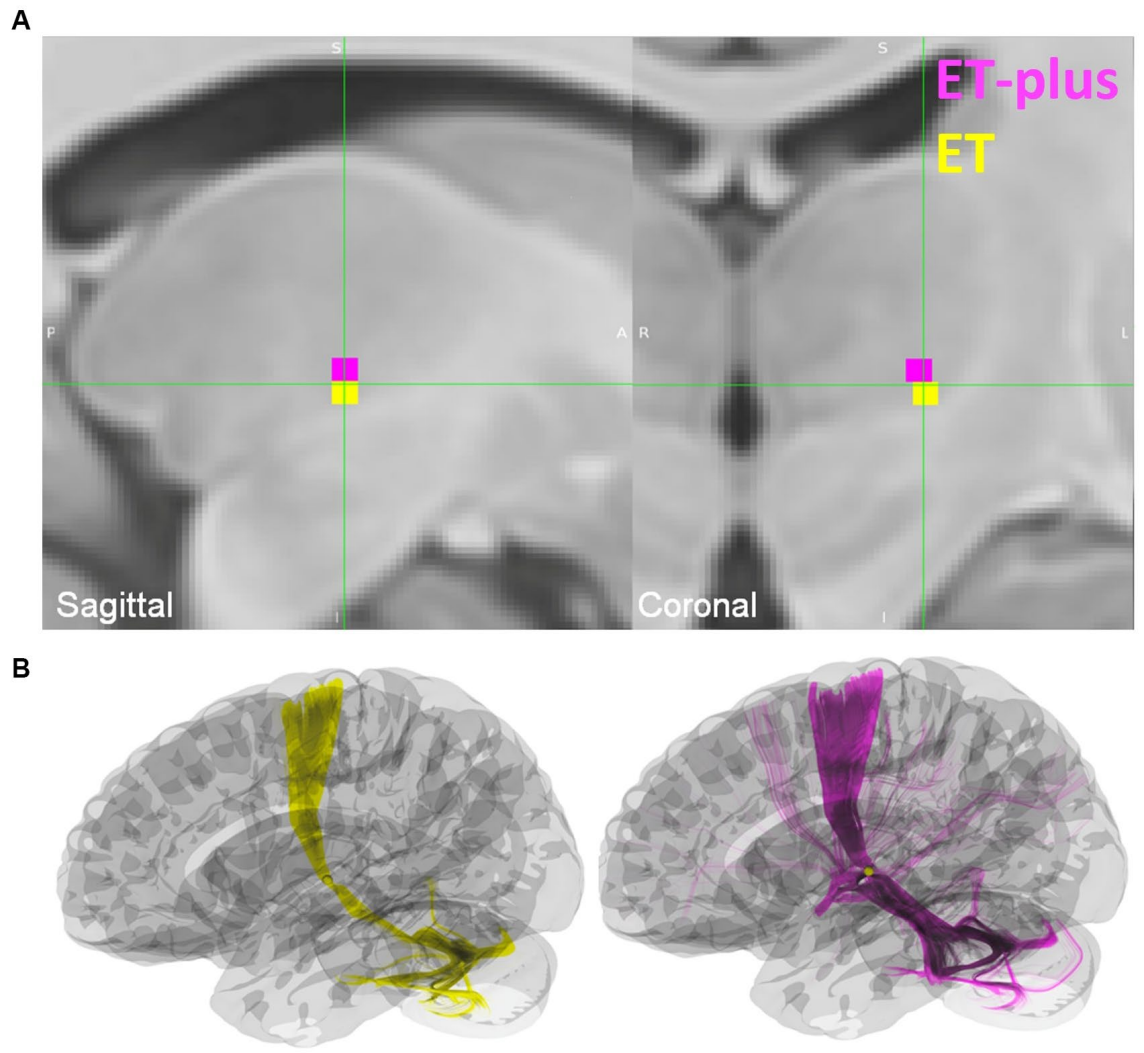
**(A)** Center of gravity (COG) analysis for ET shown in yellow, and ET-plus shown in magenta. **(B)** Location of the ET and ET-plus COG in relation to DRTT.

DBS electrode trajectories were recreated based on active contacts. Comparisons of ET and ET-plus trajectories in relation to thalamic nuclei and relevant white matter tracts are shown in Figure 1. DBS electrodes were primarily located at the VIM and ventralis oralis posterior (VOp) borders and were in close proximity to DRTT, nDRTT, and ansa lenticularis. The volume of tissue activated (VTA) for clinically optimized DBS electrodes for ET and ET-plus cohorts were created in Lead DBS as a heat map (Figure 2). The optimal stimulation region for ET and ET-plus correlated to the same region as represented in the VTA heat map. Figure 3 demonstrates the relative location of ET and ET-plus heatmap to adjacent relevant white matter tracts including DRTT, nDRTT and ansa lenticularis. For each group, a center of gravity (COG) analysis was also carried out. The COG for optimal stimulation for ET was located at MNI −13.8, −15.1, and −0.1 in the ventral VIM region, while the optimal stimulation for ET-plus was located at MNI −13.6, −15.6, and +1.3. The COG for ET-plus cohort was slightly more dorsal within the ventral VIM compared to ET cohort (Figure 4A). No significant group differences were found in VTA overlap with the DRTT (*t* = 0.375, df = 41, *p* = 0.713), nDRTT (*t* = −1.173, df = 41, *p* = 0.247) or the ansa lenticularis (*t* = 1.675, df = 41, *p* = 0.102) as shown in Figures 3, 4B. Additionally, overall VTA size did not differ between groups (*t* = −1.416, df = 41, *p* = 0.164).

**Figure 5:**
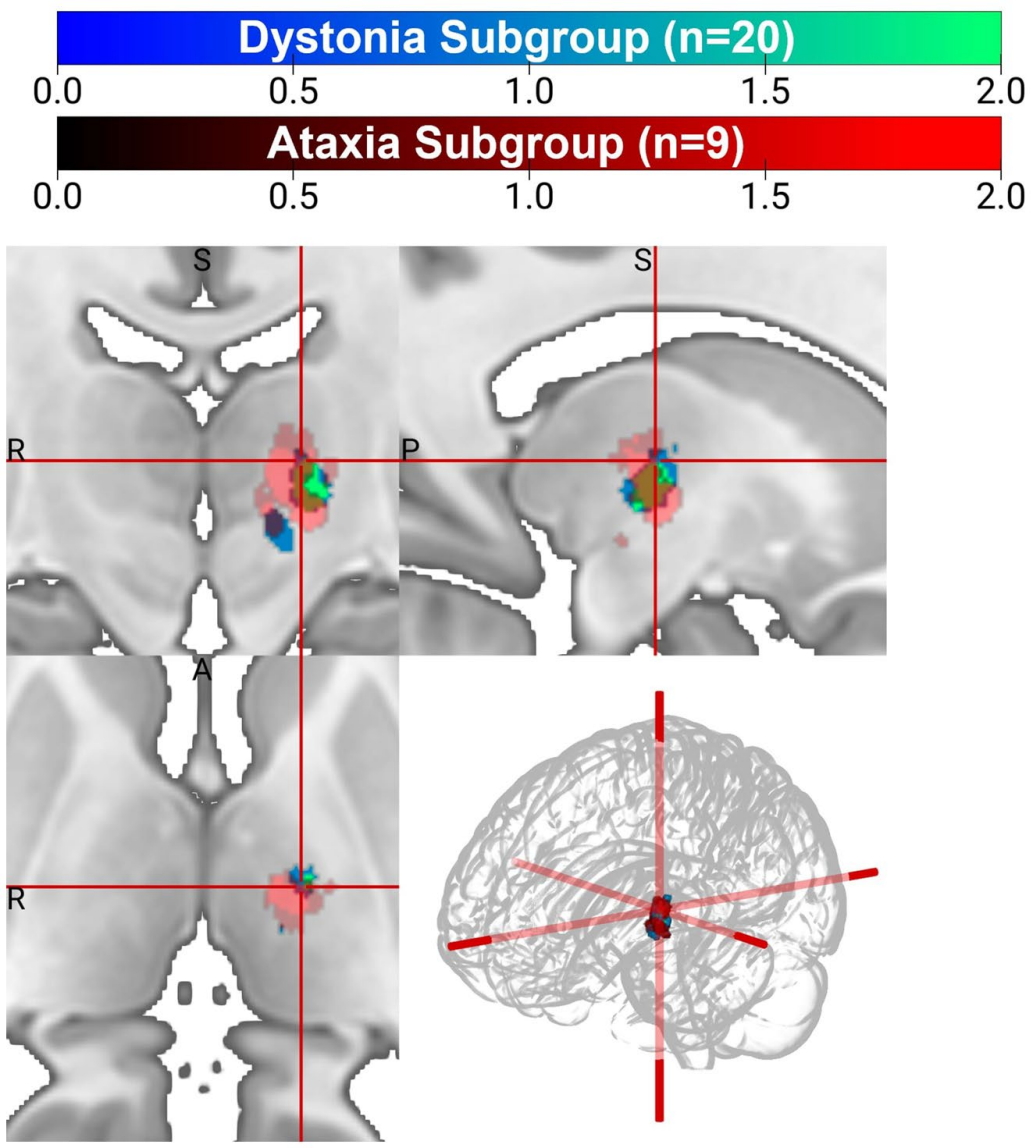
VTA heatmap for ET-plus dystonia (red) and ET-plus ataxia (blue-green).

Dystonia was the more prevalent plus feature in the ET-plus subgroups. VTA analysis for ET-plus subgroups was carried out specifically for ET-plus with dystonia (*n* = 20) and ET-plus with ataxia (*n* = 9) (Figure 5). ET-plus with dystonia has a slightly more medial optimal VTA connectivity compared to ET-plus with ataxia.

## Discussion

Our study aimed to delineate effective stimulation sites in ET and ET-plus patients, under the hypothesis that optimal VTA for each cohort would be distinctly different in the ventral thalamus, suggesting different underlying pathophysiologic mechanisms. This is the first study to further evaluate ET-plus subtypes, focusing on dystonia and ataxia as distinctly different from ET. Our cohort had more ET-plus patients compared to ET patients, with 87.5% of patients being re-classified to ET-plus, similar to recent literature reporting up to 50–83% of ET patients being reclassified to ET-plus (Prasad and Pal, 2019; Bellows and Jankovic, 2021). Our data found that VIM DBS is effective in both ET and ET-plus patients with comparable outcome, again in line with recent retrospective studies (Steffen et al., 2020; Gilmour et al., 2021). Clinically optimized VTAs were estimated to be within the same region of the ventral thalamus for ET and ET-plus patients in our data. While there were subtle differences in the ET and ET-plus COG analysis, where ET-plus COG was slightly more dorsal, presumably influenced by having more patients with dystonic features, we do not believe this result was clinically significant and should be interpreted with caution. Within ET-plus cohort, we further analyzed ET-plus with dystonia (*n* = 20), and ET-plus with ataxia (*n* = 9). ET-plus with dystonia subgroup has a more medially placed COG compared to that of ET-plus with ataxia, with unclear clinical significance. In addition, COG location relative to the DRTT showed that the VTAs for ET and ET-plus cohorts overlap significantly with this tract, which is likely the main contributor of tremor suppression as demonstrated in prior studies (Al-Fatly et al., 2019; Middlebrooks et al., 2021).

The new classification of ET-plus remains controversial as interpretations for neurologic soft signs can be subjective and the designation was not based on any neuroimaging, genetic or pathologic basis (Louis et al., 2020). A post-mortem study by Gionco et al. comparing ET and ET-plus patients did not demonstrate any pathologic differences (Gionco et al., 2021), further bringing into question whether ET and ET-plus are two distinct entities or a continuum of the same condition. Our study did not demonstrate a different optimal VTA site between ET and ET-plus, but our data further support a common tremor network between ET and ET-plus as demonstrated by Middlebrooks et al. (2021). A small matched retrospective study by Tsuboi et al. showed that dystonic tremor has an optimal connectivity between the VIM and VOp border (Tsuboi et al., 2021), related to stimulation of the pallidothalamic fibers. This finding may also explain how the VTA connectivity for ET-plus with dystonia was more medial in our subgroup analysis but there’s insufficient evidence to suggest an alternative DBS target. While the 2018 consensus classification aimed to reduce heterogeneity in ET patients, ET is widely known for being a heterogeneous condition. In addition, it is increasingly apparent that ET is a dynamic syndrome and patients often evolve to have additional plus signs as their symptoms progress (Prasad and Pal, 2019; Louis et al., 2020; Bellows and Jankovic, 2021). A major limitation of the new consensus designation is the lack of objective measures for neurologic soft signs. Our study was also limited by having only one movement disorders specialist subjectively evaluate for video evidence of additional neurologic soft signs. It can be challenging to visually discern subtle posturing due to age-related or arthritic changes from dystonic posturing. A systematic review employed Bayesian analysis to estimate the probability of a patient having ET or ET-plus showed that having two or more soft signs makes an alternative diagnosis than ET more likely (Elble, 2022). The author also proposes additional diagnostic considerations to further distinguish ET from ET mimics such as utilizing electrophysiology to rule out enhanced physiologic tremor (EPT) or employing somatosensory temporal discrimination threshold to discern ET from dystonia (Elble, 2022). Future prospective studies with larger sample size, more stringent and objective assessments of different neurologic soft signs with correlating outcome measures (i.e., ET-plus with dystonia, ET-plus with ataxia, or ET-plus with cognitive impairment) may shed more light into differentiating ET-plus subtypes.

Due to the retrospective design, our data only captured specific connectivity snapshots, with a wide range of follow-up periods considering patients who were later enrolled into the study. It is difficult to confirm whether there were subtle changes in optimal stimulation sites over time as patients’ symptoms evolve. Similarly, changes within VTA overlaps due to habituation or disease progression were not well captured in this study. A prospective, longitudinal study with evaluations at regular intervals may help us better understand the difference between ET and ET-plus, and even bring more insights into ET disease progression. Our study has additional limitations: the sample size was drastically reduced due to loss of pre-operative videos, most subjects had bilateral VIM implants, which may affect optimal stimulation parameters to avoid stimulation-induced side effects, VTAs were recreated in MNI space and unable to account for subject’s individual anatomic differences, there was no functional connectivity data available to corroborate for clinical tremor reduction, and this was not a blinded study.

## Conclusion

VIM DBS therapy is efficacious in patients with ET and ET-plus. There were no significant differences in optimal stimulation site or VTA overlap with white matter fiber tracts between ET, ET-plus and ET-plus subgroups.

## Data availability statement

The original contributions presented in the study are included in the article/Supplementary material, further inquiries can be directed to the corresponding author.

## Ethics statement

This study was approved by the Health Sciences South Carolina (HSSC) electronic Institutional Review Board (eIRB number Pro00062817). Written informed consent for participation was not required for this study in accordance with the local legislation and institutional requirements.

## Author contributions

CY: Conceptualization, Data curation, Formal analysis, Investigation, Methodology, Visualization, Writing – original draft, Writing – review & editing. DL: Data curation, Formal analysis, Investigation, Methodology, Writing – review & editing. CC: Data curation, Writing - review & editing. NR: Writing – review & editing, Resources. IT: Resources, Writing – review & editing. GR: Conceptualization, Methodology, Supervision, Writing – review & editing.

## Footnotes

^2^ http://stnava.github.io/ANTs/

^3^ http://www.fil.ion.ucl.ac.uk/spm/software/

^4^ https://fsl.fmrib.ox.ac.uk/
