# Supplementary material for: Deep brain stimulation for essential tremor versus essential tremor plus: should we target the same spot in the thalamus?
Source: Front Hum Neurosci. 2023 Oct 31;17:1271046. doi: 10.3389/fnhum.2023.1271046 (PMC10644388; doi:10.3389/fnhum.2023.1271046)
Supplement: Supplementary file 1 [file Table_1.docx]

Supplementary Table 1. Clinical characteristics of individual subject

| Patient No. | Diagnosis | Sex | Age at symptom onset  (years) | Age at DBS  (years) | More affected side | Total TRS at Baseline | Pre-DBS unilateral  TRS motor subscore | Post-DBS  unilateral  TRS motor subscore | TRS unilateral motor reduction after DBS (%) | Final Follow Up Period (months) | TRS Follow Up Period | Device | One to two-yearfollow up Stimulation parameters (Left or right implant, cathodes, anodes, voltage or milliamps, pulse width, and frequency) | Final Stimulation parameters  (Left or right implant, cathodes, anodes, voltage or milliamps, pulse width, and frequency) |
| --- | --- | --- | --- | --- | --- | --- | --- | --- | --- | --- | --- | --- | --- | --- |
| 1 | ET | F | 48 | 69 | R | 91 | 33 | 0 | 100 | 55 | 9 | Medtronic | L: 1-,2-, C+, 2.7 V, 60 uS, 130 Hz  R: 0-,1-, C+, 3.0 V, 60 uS, 170 Hz | L: 1-,2-, C+, 2.6 V, 60 uS, 130 Hz R: 0-,1-, C+, 2.4 V, 60 uS, 170 Hz |
| 2 | ET | M | 11 | 77 | R | 68 | 1 | 1 | 0 | 56 | 15 | Medtronic | L: 0-, C+, 2.9 mA, 90 uS, 180 Hz | L: 1-, C+, 3.4 mA, 80 uS, 155 Hz |
| 3 | ET | M | 37 | 60 | R | 55 | 6 | 0 | 100 | 16 | 7 | Medtronic | L: 1-, C+, 2.0 mA, 70 uS, 170 Hz R: 1-, 3+, 2.0 mA, 60 uS, 160 Hz | L: 1-, C+, 2.0 mA, 70 uS, 170 Hz R: 1-, 3+, 2.0 mA, 60 uS, 160 Hz |
| 4 | ET | M | 41 | 56 | R | 61 | 10 | 0 | 100 | 65 | 7 | Medtronic | L: 0/1-, 3+, 3.5V, 90 uS, 170 Hz  2.33 mA | L: 1-, 3+, 4.8 mA, 120 uS, 180 Hz |
| 5 | ET | F | 46 | 71 | R | 55 | 8 | 1 | 88 | 90 | 11 | Medtronic | L: 1-, 3+, 3.2V, 60 uS, 180 Hz 1.856 mA R: 1-, 2+, 2.8V, 90 uS, 180 Hz 3.4 mA | L:0-, 3+, 3.5 mA, 60 uS, 180 Hz R: 1-, 3+, 3.0 mA, 80 uS, 180 Hz |
| 6 | ET | F | 51 | 71 | L | 47 | 6 | NA | NA | 27 | NA | Medtronic | R: 2-, C+, 2.2 mA, 60 uS, 130 Hz | R: 2-, C+, 2.5 mA, 60 uS, 160 Hz |
| 7 | ET | M | 7 | 60 | R | 50 | 10 | NA | NA | 10 | 19 | Medtronic | L; 1-, C+, 3.0 V, 90 uS, 160 Hz 2.44 mA | L; 1-, C+, 3.0 V, 90 uS, 160 Hz 2.44 mA |
| 8 | ET | M | 26 | 66 | R | 53 | 3 | 1 | 67 | 86 | 6 | Medtronic | L: 2-, 3+, 3.6 V, 120 uS, 170 Hz 1.85 mA R: 1-, 3+, 3.7V, 120 uS, 170 Hz 2.0 mA | L: 2-, 3+, 4.2 V, 120 uS, 180 Hz 2.7 mA R: 1-, 3+, 4.3V, 120 uS, 180 Hz 3.2 mA |
| 9 | ET | M | 10 | 77 | R | 30 | 4 | 1 | 75 | 39 | 20 | Medtronic | L: 0-, C+, 3.5 mA, 70 uS, 180 Hz  R: 0-, 3+, 1.4 mA, 60 uS, 165 Hz | L: 1-, C+, 3.1 mA, 60 uS, 150 Hz R: OFF |
| 10 | ET | M | 65 | 74 | R | 41* | 6 | NA | NA | 25 | NA | Boston Scientific | L: L1 30%, S3 70%, C+, 1.7 mA, 60 uS, 149 Hz | L: L1 40%, S2/3 30%, C+, 4.5 mA, 60 uS, 179 Hz |
| 11 | ET + dystonia | F | 36 | 68 | L | 23 | 7 | NA | NA | 70 | NA | Medtronic | R: 2-, C+, 3.0V, 60 uS, 130Hz 2.96 mA | R: 3-, C+, 4.7 mA, 60 uS, 170 Hz |
| 12 | ET + ataxia | M | 14 | 72 | R | 19 | 8 | 5 | 38 | 33 | 13 | Boston Scientific | L: S3,4 (25%/75%), C+, 2.2mA, 70 uS, 179 Hz R: L2 (2,4), C+, 2.6 mA, 70 uS, 170 HZ | L: L2-3, C+, 3.6 mA, 90 uS, 179 Hz R: L2, C+, 3.0 mA, 90 uS, 179 Hz |
| 13 | ET + dystonia | M | 47 | 73 | R | NA | NA | NA | NA | 44 | NA | Medtronic | L: 0-, C+, 3.7 mA, 60 uS, 170 Hz R: 2-, C+, 2.9 mA, 60 uS, 130 Hz | L: 0-, C+, 4.1 mA, 80 uS, 180 Hz R: 2-, C+, 3.5 mA, 60 uS, 170 Hz |
| 14 | ET + dystonia | F | 50 | 83 | R | 53 | 6 | NA | NA | 41 | NA | Medtronic | L: 0-, 3+, 1.5V, 60 uS, 160 Hz  3.0 mA R: 2-, C+, 4.1 mA, 90 uS, 180 HZ | L: 0-, C+, 2.2 mA, 60 uS ,160 Hz R: 1-. 3+, 2.8 mA, 60 uS, 180 Hz |
| 15 | ET + dystonia | M | 19 | 69 | L | 86 | 19 | NA | NA | 7 | NA | Medtronic | L: 1-, 3+, 3.0V, 90 uS, 170 Hz 1.7mA | L: 1-, 3+, 3.0V, 90 uS, 170 Hz 1.7mA |
| 16 | ET + ataxia | M | 28 | 69 | R | 58 | 5 | 1 | 80 | 32 | 7 | Boston Scientific | L: S3/4-, C+, 2.5mA, 60uS, 170Hz R: S3/4-, C+, 2.5mA, 70uS, 170Hz | L: S3/6-, C+, 3.4 mA, 50 uS, 170 Hz R: L3-, C+, 2.3 mA, 50 uS, 170 Hz |
| 17 | ET + parkinsonism | M | 63 | 75 | R | 41 | 4 | 1 | 75 | 42 | 4 | Medtronic | L: 1-, 3+, 3.5 mA, 80 uS, 180 Hz R: 1-, 2+, 3.1 mA, 80 uS, 185 Hz | L: 1-, 3+, 3.6 mA, 60 uS, 180 Hz R: 1-, 2+, 3.0 mA, 60 uS, 185 Hz |
| 18 | ET + dystonia | F | 45 | 75 | L | 56 | 13 | 0 | 100 | 17 | 19 | Medtronic | R: 2-, C+, 2.0 mA, 60 uS, 160 Hz | R: 2-, C+, 2.0 mA, 60 uS, 160 Hz |
| 19 | ET + dystonia | M | 48 | 64 | R | 53* | 10 | NA | NA | 39 | NA | Boston Scientific | L: L3-, C+, 2.2 mA, 60 uS, 179 Hz R: L2.5 (S2 - 30%, S4 - 30%, S5 - 20%, S7 - 20%), C+, 3.0 mA, 70 uS, 179 Hz | L: L3-, C+, 3.1 mA, 70 uS, 179 Hz R: (S2- 45%, S4-15%, S5-30%, S7 -10%), 4.3 mA, 70 uS, 185 Hz |
| 20 | ET + dystonia | M | 50 | 78 | L | 49 | 6 | 2 | 67 | 35 | 40 | Medtronic | L: 1-, 0+, 3.3 V, 90 uS, 160 Hz 3.6 mA R: 2-, 1+, 3.2 V, 80 uS, 160 Hz 4 mA | L: 1-, 0+, 3.3V, 90 uS, 160 Hz 3.6 mA R: 1-, 3+, 3.5 mA, 90 uS, 180 Hz |
| 21 | ET + dystonia | M | 55 | 78 | L | 66 | 12 | 4 | 67 | 30 | 19 | Boston Scientific | R: L3 (focus on S7 64%, S5/6 18%), C+, 2.6 mA, 60 uS, 174 Hz | R: L3 75%, L2 25%, C+, 4.9 mA, 80 uS, 174 Hz |
| 22 | ET + dystonia | M | 20 | 58 | L | 40 | 5 | 5 | 0 | 26 | 9 | Abbott | R: 3C-, C+, 2.5 mA, 80 uS, 180 Hz | R: 2C/3C-, C+, 3.1 mA, 80uS, 180 Hz |
| 23 | ET + dystonia | M | 50 | 65 | R | 53 | 11 | 0 | 100 | 60 | 11 | Medtronic | L:1-, C+, 3.4 mA, 80 uS, 160 Hz R: 1-, C+, 2.0 mA, 60 uS, 160 Hz | L: 1-, C+, 3.5 mA, 120 uS, 180 Hz R: 2-, C+, 3.3 mA, 70 uS, 180 Hz |
| 24 | ET + dystonia | F | 18 | 68 | L | 47 | 13 | 3 | 77 | 42 | 3 | Medtronic | L: 2-, C+, 2.8 mA, 60 uS, 160 Hz R: 2-, 3+, 3.0 mA, 80 uS, 170 Hz | L: 1-, 3+, 3.7 mA, 70 uS, 180 Hz R: 1-, 3+, 3.9 mA, 100 uS, 180 Hz |
| 25 | ET + ataxia | F | 50 | 74 | R | 74 | 17 | NA | NA | 59 | NA | Medtronic | L: 1-, C+, 3.3V, 120 uS, 160 Hz 2.2 mA | L: 0-1-, C+, 2.4V, 130 uS, 160 Hz 3.3 mA |
| 26 | ET + dystonia | F | 15 | 65 | R | 59 | 10 | 3 | 70 | 70 | 19 | Medtronic | L: 1-, 3+, 3.0 mA, 60 uS, 160 Hz R: 1-, C+, 3.2 V, 60 uS ,160 Hz 3.45 mA | L: 0-, C+, 3.7 mA, 60 uS, 180 Hz R: 0-, C+, 3.7 mA, 70 uS, 180 Hz |
| 27 | ET + dystonia | F | 66 | 76 | R | 55 | 9 | 3 | 67 | 32 | 7 | Abbott | L: 2C-, C+, 2.9 mA, 70 uS, 180 Hz | L: 2BC-, C+, 3.0 mA, 80 uS, 180 Hz |
| 28 | ET + dystonia | M | 35 | 79 | L | 87 | 20 | 2 | 90 | 35 | 4 | Boston Scientific | NA | L: L2-, C+, 2.7 mA, 60 uS, 170 Hz R: L2-, C+, 2.5 mA, 60 uS, 170 Hz |
| 29 | ET + ataxia | F | 55 | 66 | R | 49 | 7 | NA | NA | 27 | NA | Boston Scientific | L: L3-, C+, 2.6 mA, 80 uS, 185 Hz R: L2-, C+, 2.2 mA, 70 uS, 185 Hz | L: S3-, C+, 1.7 mA, 60 uS, 159 Hz R: L2-, C+, 3.5 mA, 70 uS, 159 Hz |
| 30 | ET + ataxia | F | 57 | 69 | L | 76 | NA | 1 | NA | 7 | NA | Boston Scientific | L: S2/4/5/7 (25%), 2.0 mA, 50 uS, 154 Hz R: S6/7 (50/50%), 3.5 mA, 60 uS, 154 Hz | L: S2/4/5/7 (25%), 2.0 mA, 50 uS, 154 Hz R: S6/7 (50/50%), 3.5 mA, 60 uS, 154 Hz |
| 31 | ET + dystonia | F | 10 | 71 | R | 42* | 17 | 5 | 71 | 5 | 13 | Boston Scientific | L: L3-, C+, 2.7 mA, 60 uS, 149 Hz | L: L3-, C+, 2.7 mA, 60 uS, 149 Hz |
| 32 | ET + dystonia | F | 73 | 73 | R | 53 | 7 | 3 | 57 | 1 | 8 | Boston Scientific | L: S2/S3 (75%/25%), C+, 3.4 mA, 50 uS, 170 Hz | L: S2/S3 (75%/25%), C+, 3.4 mA, 50 uS, 170 Hz |
| 33 | ET + dystonia | M | 60 | 75 | R | 35 | 7 | 2 | 71 | 3 | 9 | Medtronic | L: 1a- (0.8 mA), 1c- (0.5 mA), 2a- (1.0 mA), C+, 2.9 mA, 40 uS, 170 Hz | L: 1a- (0.8 mA), 1c- (0.5 mA), 2a- (1.0 mA), C+, 2.9 mA, 40 uS, 170 Hz |
| 34 | ET + dystonia | M | 15 | 74 | L | NA | 7 | 1 | 86 | 1 | 4 | Abbott | R: 11BC-, 11A+, 2.8 mA, 60 uS, 180 Hz | R: 11BC-, 11A+, 2.8 mA, 60 uS, 180 Hz |

DBS = deep brain stimulation; ET = essential tremor; TRS = Fahn-Tolosa-Marin Tremor Rating Scale; TRS unilateral motor subscore of the more affected limb included Items 1-5, 7-8 for right hand or items 1-4, 7 and 9 for left hand

*indicates incomplete TRS, and these data points were not included in statistical analyses.

For Boston Scientific devices: L indicates ring mode at level number 1-4, and S indicates a segmental lead.

Supplemental Table 2. Clinical characteristics for ET-plus cohort

| Patient No. | Diagnosis | Sex | ET-plus feature |
| --- | --- | --- | --- |
| 11 | ET + dystonia | F | Subtle cervical dystonia |
| 12 | ET + ataxia | M | Wide based gait |
| 13 | ET + dystonia | M | Cervical dystonia with dystonic head tremor |
| 14 | ET + dystonia | F | Dystonic posturing in limb |
| 15 | ET + dystonia | M | Multidirectional head tremor |
| 16 | ET + ataxia | M | Gait ataxia and dysarthria |
| 17 | ET + parkinsonism | M | Non-progressive parkinsonism |
| 18 | ET + dystonia | F | Multidirectional head tremor and dystonic posturing in limb |
| 19 | ET + dystonia | M | Subtle cervical dystonia |
| 20 | ET + dystonia | M | Subtle cervical dystonia |
| 21 | ET + dystonia | M | Mirror movements and subtle limb posturing, tremor varies with changing position |
| 22 | ET + dystonia | M | Dystonic posturing in limb |
| 23 | ET + dystonia | M | Subtle cervical dystonia |
| 24 | ET + dystonia | F | Subtle limb posturing |
| 25 | ET + ataxia | F | Wide based and cautious gait |
| 26 | ET + dystonia | F | Subtle cervical dystonia with multidirectional head tremor |
| 27 | ET + dystonia | F | Subtle cervical dystonia |
| 28 | ET + dystonia | M | Mirror movements with dystonic limb posturing bilaterally |
| 29 | ET + ataxia | F | Difficulty with tandem gait |
| 30 | ET + ataxia | F | Mildly wide based gait |
| 31 | ET + dystonia | F | Subtle cervical and limb posturing |
| 32 | ET + dystonia | F | Subtle cervical dystonia |
| 33 | ET + dystonia | M | Subtle limb posturing with positional dependent tremor |
| 34 | ET + dystonia | M | Subtle limb posturing with positional dependent tremor |
